# Non-Muscle Myosin II Is Essential for the Negative Regulation of B-Cell Receptor Signaling and B-Cell Activation

**DOI:** 10.3389/fimmu.2022.842605

**Published:** 2022-04-14

**Authors:** Margaret K. Seeley-Fallen, Michelle Lazzaro, Chaohong Liu, Quan-Zhen Li, Arpita Upadhyaya, Wenxia Song

**Affiliations:** ^1^Department of Cell Biology & Molecular Genetics, University of Maryland, College Park, MD, United States; ^2^Department of Immunology and Internal Medicine, University of Texas Southwestern Medical Center, Dallas, TX, United States; ^3^Department of Physics, University of Maryland, College Park, MD, United States

**Keywords:** B lymphocytes, B-cell receptor, actin cytoskeleton, non-muscle myosin II, signal transduction, antibody response

## Abstract

Antigen (Ag)-triggered B-cell receptor (BCR) signaling initiates antibody responses. However, prolonged or uncontrolled BCR signaling is associated with the development of self-reactive B-cells and autoimmune diseases. We previously showed that actin-mediated B-cell contraction on Ag-presenting surfaces negatively regulates BCR signaling. Non-muscle myosin II (NMII), an actin motor, is involved in B-cell development and antibody responses by mediating B-cell migration, cytokinesis, and Ag extraction from Ag-presenting cells. However, whether and how NMII regulates humoral responses through BCR signaling remains elusive. Utilizing a B-cell-specific, partial NMIIA knockout (cIIAKO) mouse model and NMII inhibitors, this study examined the role of NMII in BCR signaling. Upon BCR binding to antibody-coated planar lipid bilayers (PLB), NMIIA was recruited to the B-cell contact membrane and formed a ring-like structure during B-cell contraction. NMII recruitment depended on phosphatidylinositol 5-phosphatase (SHIP1), an inhibitory signaling molecule. NMII inhibition by cIIAKO did not affect B-cell spreading on PLB but delayed B-cell contraction and altered BCR clustering. Surface BCR “cap” formation induced by soluble stimulation was enhanced in cIIAKO B-cells. Notably, NMII inhibition by cIIAKO and inhibitors up-regulated BCR signaling in response to both surface-associated and soluble stimulation, increasing phosphorylated tyrosine, CD79a, BLNK, and Erk and decreasing phosphorylated SHIP1. While cIIAKO did not affect B-cell development, the number of germinal center B-cells was significantly increased in unimmunized cIIAKO mice, compared to control mice. While cIIAKO mice mounted similar antibody responses when compared to control mice upon immunization, the percentages of high-affinity antibodies, Ag-specific germinal center B-cells and isotype switched B-cells were significantly lower in cIIAKO mice than in control mice. Furthermore, autoantibody levels were elevated in cIIAKO mice, compared to control mice. Collectively, our results reveal that NMII exerts a B-cell-intrinsic inhibition on BCR signaling by regulating B-cell membrane contraction and surface BCR clustering, which curtails the activation of non-specific and self-reactive B-cells.

## Introduction

B-cells are responsible for mounting antibody responses, which neutralize pathogens and flag pathogens for immune clearance. B-cells express clonally specific receptors, the B-cell receptor (BCR), which senses foreign antigens (Ag). Ag binding of the BCR initiates the activation of naïve mature B-cells by inducing signaling cascades (1–4). BCR signaling activates transcriptional programs for B-cell proliferation and differentiation and promotes Ag internalization, processing, and presentation to acquire T-cell help (5–8). B-cell activation and differentiation lead to germinal center formation, where B-cells undergo somatic hypermutation and affinity maturation. B-cells compete for Ag in an affinity-dependent manner, triggering and modulating signaling and Ag presentation to T-cells. Both BCR signaling and T-cell help are essential for B-cells to differentiate into high-affinity memory B-cells and antibody-secreting cells (4, 9, 10). Furthermore, BCR-initiated B-cell activation is tightly regulated to control the quality, quantity, and duration of antibody responses based on the nature of antigenic challenges (11, 12). Dysregulation of this process, such as prolonged or constitutive BCR activation, can lead to the activation of self-reactive B-cells and autoantibody production, as well as B-cell malignancies (13–16). Thus, understanding the mechanisms underlying BCR signaling regulation is essential for developing vaccines for pathogens and therapies for autoimmune diseases and cancer.

BCR signaling is initiated by receptor oligomerization or clustering on the B-cell surface, induced by soluble multivalent Ag as well as insoluble surface-associated Ag (17–19). Receptor clustering leads to the association of BCRs with lipid rafts, enabling the phosphorylation of tyrosine-based immune receptor activation motifs in the cytoplasmic domains of the BCR by raft-resident Src kinases (20, 21). Phosphorylated BCRs recruit and activate the Src-like kinase Syk and the adaptor protein BLNK, initiating signaling cascades that include phospholipase Cγ2 (PLCγ2), phosphatidylinositol-3 kinase (PI3K), and the small GTP-binding protein Ras, which activate Ca^2+^ flux, protein kinase C, and MAP kinases (1, 2, 5). BCR clustering can also trigger the formation of supramolecular activation complexes at one pole of the B-cell or at the B-cell membrane region contacting Ag-presenting surfaces (called the immunological synapse), depending on the affinity, valency, concentration, and density of Ag (3, 22, 23). For naïve mature B-cells, BCR signaling is relatively transient. BCR signaling also triggers inhibitory phosphatases, such as the tyrosine phosphatase SHP1 and phosphatidylinositol-5 phosphatase SHIP1, which down-regulate signaling (24, 25). Mutations that impair the function of stimulatory kinases in the BCR signaling pathway can cause B-cell and humoral immune deficiencies (26, 27), while B-cell-specific deletion of SHP1 or SHIP1 leads to increases in autoantibody production (24, 25). However, the mechanism by which the nature of encountered Ag regulates the balance between BCR signaling activation and deactivation remains elusive.

The actin cytoskeleton is an important regulator of BCR signaling (28–30). BCR clustering on the surface of resting B-cells requires disassembly of the cortical F-actin network that limits BCR lateral mobility on the plasma membrane (31, 32). Previous studies have shown that pharmacologically depolymerizing F-actin triggers BCR signaling (31). Following transient F-actin depolymerization, actin rapidly polymerizes, which promotes surface BCR clustering and polarization of clusters to one pole of the cell, amplifying signaling in response to soluble Ag (33). In addition to promoting BCR clustering and movement of surface BCRs to the B-cell membrane region interacting with surface-associated Ag (contact zone), rapid actin polymerization drives B-cell spreading, expanding the interaction area of B-cells with Ag-presenting surfaces (33, 34). Inhibition of this rapid actin polymerization by deletion of Bruton’s tyrosine kinase (Btk), a signaling molecule that activates actin polymerization, or the downstream actin nucleation promoting factors, Wiskott-Aldrich Syndrome protein (WASp) and Neuronal WASp (N-WASp), blocks B-cell spreading and signal amplification (35, 36). Subsequent to spreading, B-cells undergo contraction, which reduces the B-cell contact zone and consequently drives BCR clusters to merge (34, 36, 37). We have previously shown that the contraction is critical for BCR signaling attenuation. B-cell-specific deletion of the inhibitory phosphatase SHIP1 and the actin nucleation promoting factor N-WASp and germline deletion of actin-binding protein 1 (Abp1) all delay B-cell contraction and lead to prolonged BCR signaling and autoantibody production (35–37). However, how the actin cytoskeleton coordinates with inhibitory signaling molecules to negatively regulate BCR signaling is not well understood.

The actin motor protein, non-muscle myosin II (NMII), is known to mediate plasma membrane contraction during cell migration and cytokinesis in most mammalian cell types (38), consistent with a potential role for NMII in Ag-induced B-cell contraction. Using CD79a (*mb1*)-Cre-driven B-cell specific knockout of NMIIA, the predominant isoform of NMII expressed in B-cells, and CD23-Cre-driven NMII knockout mouse models, Hoogeboom *et al*. confirmed the role of NMII in B-cell proliferation and migration (39). That study also showed that NMIIA-deficient follicular B-cells exhibit elevated levels of activation markers, implying NMII in the negative regulation of B-cell activation (39). NMII has also been shown to be essential for B-cells to capture Ag from presenting surfaces *in vitro* and *in vivo*  (40). Importantly, Ag capture initiates Ag processing and presentation. NMII-driven traction forces pull Ag from presenting surfaces, enabling B-cells to acquire Ag based on BCR binding affinity (40–42). We have recently shown that NMII mediates antigen capture by causing B-cell membrane permeabilization, which induces a membrane repair response involving lysosome exocytosis (43). NMIIA-deficiency ultimately causes impaired antibody responses against antigenic challenges (39). However, whether and how NMII regulates BCR signaling and subsequent B-cell responses remains unclear due to its contribution to multiple aspects of cellular functions.

Here, we examined the role of NMII in BCR signaling utilizing GFP-NMIIA transgenic mice and a partial B-cell-specific NMIIA knockout mouse model (cIIAKO), which allows normal B-cell development in the bone marrow and the periphery. We found that NMIIA is recruited to the B-cell membrane region contacting stimulatory surfaces in a SHIP1-dependent manner. Inhibition of NMII in cIIAKO animals or with inhibitors delays B-cell contraction without affecting B-cell spreading and enhances the BCR signaling response to both soluble and surface-associated stimulation. Further, cIIAKO mice display increased numbers of spontaneous germinal center B-cells and elevated levels of autoantibodies, while mounting T-dependent antibody responses with impaired affinity and memory development. Our results suggest an intrinsic role for NMII in the negative regulation of BCR signaling that is essential for the selection of high-affinity antigen-specific B-cells.

## Materials and Methods

### Mice and Cell Culture

C57BL/6J and CD19^Cre/+^ [B6.129P2(C)-Cd19^tm1(cre)Cgn^/J] mice were purchased from the Jackson Laboratory. Myh9^flox/flox^ (B6.129S‐Myh9^tm5Rsad^/Mmnc) mice were purchased from Mutant Mouse Regional Resource Center at the Jackson Laboratory. GFP-NMIIA mice were a gift of Dr. Robert Adelstein at National Heart, Lung, and Blood Institute, USA, and B-cell specific SHIP1 knockout mice (CD19^Cre/+^SHIP1^flox/flox^) were provided by Dr. Silvia Bolland at National Institute of Allergy and Infectious Diseases, USA. B-cell specific NMIIA knockout (cIIAKO) (CD19^Cre/+^Myh9^flox/flox^) mice were generated by breeding Myh9^flox/flox^ with CD19^Cre/+^ mice. CD19^+/+^Myh9^flox/flox^ mice from the breeding were used as floxed littermate control mice. Primary B-cells were isolated from the spleens of 6- to 8-week-old mice. Single-cell suspensions of splenocytes were subjected to density-gradient centrifugation in Ficoll (Sigma-Aldrich) to obtain mononuclear cells, treated with anti-Thy1.2 mAb (Biolegend) and guinea pig complement (Rockland Immunochemicals) to remove T-cells, and panned for 1 h to remove monocytes and dendritic cells. Bone marrow was flushed from mouse femurs with PBS, supplemented with 3% FBS, and subjected to density-gradient centrifugation in Ficoll (44). All animal work was reviewed and approved by the Institutional Animal Care and Usage Committee of the University of Maryland.

### RT-PCR

RNA was extracted from floxed control and cIIAKO B-cells, and cDNA corresponding to a 130 bp sequence within exon 3 of Myh9 was generated using a BioRad iScript Select cDNA Synthesis kit (BioRad). cDNA was amplified by PCR, resolved by agarose gels, and visualized by ethidium bromide.

### Preparation of Mono-Biotinylated Fab’ Antibody

Mono-biotinylated Fab’ fragment of anti-mouse antibody (Ab) (mB-Fab’-anti-IgG+M) was generated from the F(ab’)_2_ fragment (Jackson ImmunoResearch) using a published protocol (45). The disulfide bond that links the two Fab’ fragments was reduced using 2-mercaptoethylamine, and the reduced cysteine was biotinylated by maleimide-activated biotin (Thermo Scientific). Fab’ was further purified using Amicon Ultra centrifugal filters (Millipore). One biotin per Fab’ was confirmed using a biotin quantification kit (Thermo Scientific). Fab’ was labeled with Alexa Fluor 546 (AF546-mB-Fab’-anti-IgG+M) based on the manufacturer-recommended protocol (Invitrogen).

### Preparation of Planar Lipid Bilayers (PLB)

Liposome was made *via* sonication of 1,2-dioleoyl-sn-glycero-3-phosphocholine and 1,2-dioleoyl-sn-glycero-3-phosphoethanolamine-cap-biotin (Avanti Polar Lipids) in a 100:1 molar ratio. Coverslip chambers (Thermo Scientific) were coated with the PLB by incubating with the liposomes (0.05 mM) for 10 min. After extensive washes, the coated coverslip chamber was incubated with 1 µg/ml streptavidin (Jackson ImmunoResearch), followed by 1 µg/ml AF546-mB-Fab’–anti-IgG+M mixed with 9 µg/ml mB-Fab’–anti-IgG+M Ab (Fab’-PLB). For a negative control, surface BCRs were labeled with AF546-Fab’–anti-IgG+M (10 μg/ml) on ice for 30 min. The labeled B-cells were then incubated with biotinylated holo-transferrin (Tf; 16 μg/ml, an equal molar concentration of 10 μg/ml mB-Fab′–anti-Ig+M; Sigma-Aldrich) tethered to PLB by streptavidin (Tf-PLB).

### Total Internal Reflection Fluorescence Microscopic (TIRF) Analysis

Images were acquired using a Nikon TIRF system on an inverted microscope (Nikon TE2000-PFS, Nikon Instruments Inc.) equipped with a 60X, NA 1.49 Apochromat TIRF objective (Nikon), a Coolsnap HQ2 CCD camera (Roper Scientific), and two solid-state lasers of wavelength 491 and 561 nm or a DeltaVision Elite Deconvolution TIRF system on an inverted microscope (Olympus IX71 Inverted, Olympus Corporation of the Americas) equipped with a 63X, NA 1.49 TIRF objective, a sCMOS camera (Andor/Oxford Instruments), and 488 and 561 nm laser lines. Interference refection (IRM), AF488, and AF546 images were acquired sequentially. To image intracellular molecules, B-cells were incubated with Fab’-PLB at 37°C for varying lengths of time. Cells were then fixed with 4% paraformaldehyde, permeabilized with 0.05% saponin, and stained for NMIIA (Abcam), phosphorylated myosin light chain (pMLC) (S19) (Cell Signaling Technology), phosphorylated CD79a (Y182) (Cell Signaling Technology), phosphorylated SHIP1 (Y1020) (Cell Singling Technology), and phosphotyrosine mAb 4G10 (EMD Millipore). Total (TFI) and mean fluorescence intensity (MFI) of each signaling molecule and their phosphorylated forms at the B-cell contact zone as well as the B-cell contact area were determined using NIH ImageJ or custom codes written in MATLAB (The MathWorks, Natick). Background fluorescence or fluorescence generated by secondary Ab was subtracted. B-cells exhibiting NMII ring-like structures were first identified by fluorescence intensity line profiles and then visual inspection. The percentages of B-cells with NMII ring-like structures per independent experiment were quantified. For each set of data, 30~50 individual cells from three independent experiments were analyzed for each time point and condition.

### Inhibitors

Cells were treated for 20 min before and during activation with 50 µM of the NMII ATPase inhibitor Blebbistatin (Calbiochem) (46) or 10 μM of the Rho-associated protein kinase (ROCK) Y-27632 (Calbiochem) (47). Cells treated with DMSO alone were used as vehicle control (Con).

### Flow Cytometry Analysis

To analyze B-cell sub-populations, cell suspensions from the bone marrow or spleens were incubated with FcR blocking Ab (anti-mouse CD16/CD32, BD Bioscience) for 10 min on ice and stained at optimal dilutions of conjugated Abs in PBS supplemented with 1% FBS. Anti-mouse Abs and reagents used to stain bone marrow B-cells included Pacific Blue-anti-CD24 (BioLegend), biotinylated anti-Ly-51 (BP-1), streptavidin-PE, FITC-anti-CD43, PerCP-Cy5.5-anti-B220, and APC-anti-IgM (BD Biosciences). Anti-mouse Abs and reagents used to stain T1, T2, FO, and IS splenic B-cells included biotinylated-anti-IgD (Southern Biotech), AF405-streptavidin (Life Technologies), PerCP-Cy5.5-anti-B220, and FITC-anti-IgM (BD Biosciences). Anti-mouse Abs and reagents used to stain splenic MZ B-cells included Pacific Blue-anti-CD21 (BioLegend), PerCP-Cy5.5-anti-B220, and PE-anti-CD23 (BD Biosciences). Anti-mouse Abs and reagents to stain splenic germinal center (GC) B-cells included biotinylated-anti-CD95 (BD Biosciences), AF488-streptavidin (Invitrogen), PE-anti-GL7, and PerCP-Cy5.5-anti-B220 (BD Bioscience).

To analyze intracellular signaling molecules, splenic B-cells were incubated with FcR blocking Ab (BD Bioscience) for 10 min on ice, stained with APC-anti-mouse CD19 Ab (BD Biosciences) to label B-cells, washed, and activated with 10 µg/ml F(ab’)_2_ goat anti-mouse IgG+M (Jackson ImmunoResearch). Cells were then fixed, permeabilized, and stained for phosphotyrosine (pY) mAb 4G10 (EMD Millipore), pBLNK (Y84) (Santa Cruz Biotechnology), pERK (T202 Y204) (Cell Signaling Technologies), or pMLC (S19) (Cell Signaling Technologies), followed by fluorescently conjugated secondary Abs. To examine the expression levels of signaling molecules, splenic B-cells were incubated with FcR blocking Ab (anti-mouse CD16/CD32, BD Bioscience) for 10 min on ice, stained with APC-anti-mouse CD19 Ab (BD Biosciences) to label B-cells, washed, fixed, permeabilized, and labeled with Abs specific for CD79a (Cell Signaling Technologies), BLNK (Santa Cruz Biotechnology), ERK (Cell Signaling Technologies), and SHIP1 (Santa Cruz Biotechnology), followed by fluorescently conjugated secondary Abs. Cells were analyzed using a FACS Canto II flow cytometer (BD Biosciences) and FACS Diva (BD Biosciences) and FlowJo software (Tree Star).

To analyze the percentages of NP^+^ isotype switched B-cells and GC B-cells, splenic B-cells from NP-KLH immunized mice (see below) were incubated with FcR blocking Ab (BD Bioscience) for 10 min on ice, then stained with biotinylated-anti-IgD (Southern Biotech), AF405-streptavidin (Life Technologies), PerCP-Cy5.5-anti-B220, FITC-anti-IgM, PE-anti-CD138 (BD Biosciences), and APC-NP for isotype switched B-cells or biotinylated-anti-CD95 (BD Biosciences), AF488-streptavidin (Invitrogen), PE-anti-GL7, PerCP-Cy5.5-anti-B220 (BD Bioscience), and APC-NP for GC B-cells.

### BCR Capping

Surface BCR capping was analyzed using immunofluorescence microscopy. Splenic B-cells were stained with 5 µg/ml Cy3-Fab-goat-anti-mouse IgM (Jackson ImmunoResearch) for 20 min on ice, washed, activated with 10 µg/ml F(ab’)_2_ donkey anti-mouse IgG+M (Jackson ImmunoResearch) at 37°C for the indicated times, fixed, and imaged using a Zeiss LSM 980 Airyscan confocal microscope equipped with a 63X 1.4 NA oil objective. Fifteen images were randomly acquired per time point from each of three independent experiments. The percentage of B-cells displaying BCR caps for each time point was quantified by visual inspection.

### Ca^2+^ Analysis

Intracellular Ca^2+^ flux was measured by flow cytometry using the Ca^2+^ dyes Fluo4 AM and Fura Red (Molecular Probes) and manufacturer-recommended protocols. Splenic B-cells were incubated with 2 µg/ml Fluo4 and 5 µg/ml Fura Red for 30 min at 37°C, washed, and analyzed using a FACS Aria II flow cytometer (BD Biosciences). The basal fluorescence intensity of Fluo4 AM and Fura Red were measured for 60 s before stimulation with 10 µg/ml F(ab’)_2_ goat anti-mouse IgG+M (Jackson ImmunoResearch) at 37°C. The fluorescence intensity of Fluo4 AM and Fura Red was then measured for 300 s after the stimulation. The relative levels of intracellular Ca^2+^ were determined by a ratio of Fluo4 AM to Fura Red emission values using FlowJo software (Tree Star).

### Immunohistochemistry

Spleen sections from cIIAKO and floxed control mice were embedded in O.C.T Compound (Sakura Finetek) and frozen in liquid nitrogen. Sections (10 µm) were collected using a cryostat. Samples were fixed in acetone, blocked with goat serum (Jackson ImmunoResearch) and avidin/biotin (Sigma Aldrich), and then stained with optimal concentrations of the following Abs/reagents: biotinylated-anti-IgD (Southern Biotech), AF405-streptavidin (Life Technologies), AF488-anti-Thy1.2 (eBioscience), and AF647-PNA (Life Technologies). Spleen sections were imaged using a Zeiss LSM 710 Confocal Microscope (Carl Zeiss Microscopy). GC PNA MFI was measured using Zen Lite 2011 Software (Carl Zeiss Microcopy).

### Immunizations and ELISA

To analyze T cell–dependent Ab response, mice were immunized by i.p. injection of 4-hydroxy-3-nitrophenyl)acetyl–keyhole limpet hemocyanin (NP-KLH, 40 μg/mouse) (Biosearch Technologies) in Sigma Adjuvant System (Sigma Aldrich). Mice were boosted on day 28 with a second injection of NP-KLH. To determine anti-NP Ab titers in response to NP-KLH immunization, NP-BSA (Biosearch Technologies) coated ELISA plates were incubated with diluted serum, followed by HRP-conjugated anti-mouse Abs, including anti-IgM or a cocktail of anti-IgG1, IgG2a, IgG2b, and IgG3 (Southern Biotech). HRP was detected using OptEIA TMB substrate (BD Biosciences). The samples were run in triplicate and corrected for background binding. Total serum Abs were analyzed by coating ELISA plates directly with diluted serum, followed by HRP-conjugated anti-mouse Abs. Relative NP-binding affinity of serum IgG was assessed as the concentration ratio of IgG bound to NP_4_-BSA- versus NP_30_-BSA 7 days after the first and second immunization. ELISA plates were coated with NP_4_-BSA or NP_30_-BSA to capture high-affinity or total NP-specific IgG. ELISA was performed as described above.

### Autoantigen Microarray

Serum samples from 12 month-old unimmunized cIIAKO or floxed control mice were collected and processed by the Genomic and Microarray Core Facility of the University of Texas Southwestern Medical Center. In brief, 16-pad nitrocellulose film slides (Grace BioLabs) printed with 128 autoantigens or control proteins were incubated with DNase I-treated mouse serum samples at 1:50 dilutions or PBS. The Ab bound to the autoantigens on the array were detected with Cy3 goat anti-mouse IgG (H+L) and Cy5 goat anti-mouse IgM (H+L) (Jackson ImmunoResearch) and a GenePix 4400A Microarray Scanner. Images were analyzed with GenePix Pro-7.0 software. The net fluorescent intensity (NFI) of Ab binding each autoantigen was obtained by subtracting the local background and PBS controls. The signal-to-noise ratio (SNR) was calculated for Ab binding to each autoantigen. Heatmaps were generated using GraphPad’s Prism Software.

### Statistical Analysis

Statistical analysis was performed using the Student’s *t*-test (Excel, Microsoft). The statistical differences between groups indicated with *p*-values in the related graphs as: * *p*<0.05; ** *p*<0.01; *** *p*<0.001.

## Results

### B-Cell Development Is Unaffected, While Spontaneous Germinal Center B-Cells Are Increased in CD19-Cre-Driven Non-Muscle Myosin IIA Knockout (cIIAKO) Mice

**Figure 1:**
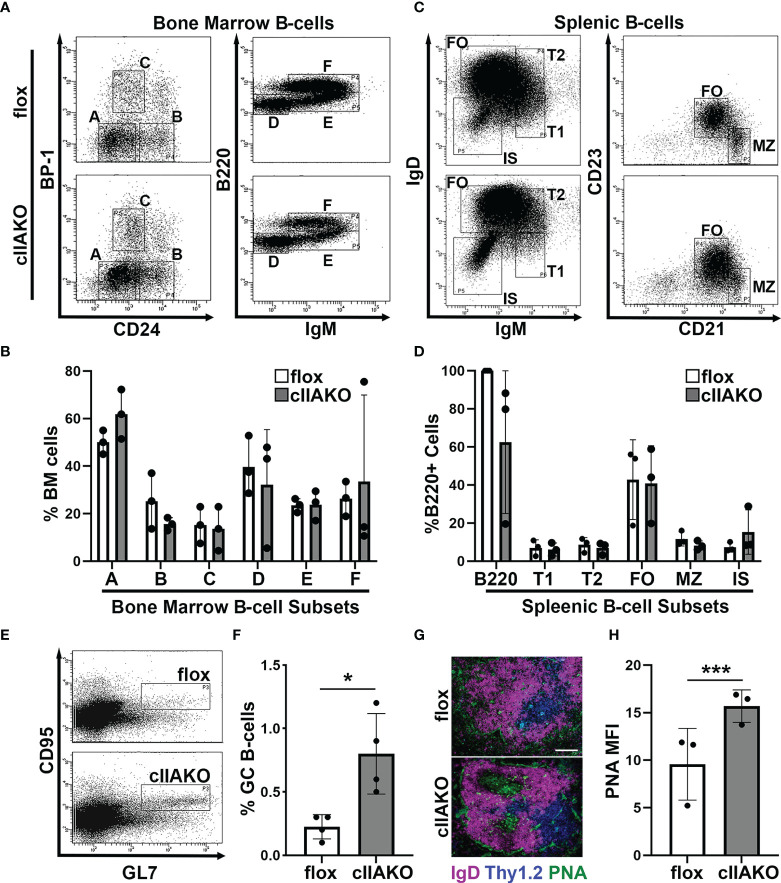
CD19 Cre-driven non-muscle myosin IIA knockout (cIIAKO) increases spontaneous germinal center (GC) B-cells without affecting B-cell development. **(A–D)** B-cell development in cIIAKO and floxed control mice. Cells from the bone marrow **(A, B)** and spleens **(C, D)** of floxed control and cIIAKO mice were labeled for surface markers of pre-pro- **(A)**, pro- **(B)**, early pre- **(C)**, late pre- **(D)**, immature **(E)** and re-circulating mature B-cells **(F)** in the bone marrow, and transitional 1 (T1), transitional 2 (T2), follicular (FO), marginal zone (MZ) and isotype switched (IS) B-cells in the spleen, and analyzed by flow cytometry. Shown are representative dot plots **(A, C)**, the average percentages (± SD) of total cells extracted from bone marrow **(B)**, and the average percentages (± SD) of total B220^+^ B-cells from the spleen **(D)**. **(E, F)** GC B-cells. Splenocytes from unimmunized mice were stained for B220, followed by the GC B-cell markers CD95 and GL7 and analyzed by flow cytometry. Shown are representative dot plots **(E)** and quantification of the mean percentage (± SD) of GC B-cells among total B220 B-cells by flow cytometry **(F)**. **(G)** Immunofluorescent staining of splenic sections from cIIAKO and floxed control mice (6-8 week old). Shown are representative follicles. Scale bar, 100 μm. **(H)** PNA MFI (± SD) in GCs per field of view (~5 images per 12 sections from each of 3 floxed control or cIIAKO mice). Data points represent individual mice **(B, D, F, H)**. n=3~4. *p<0.05, ***p<0.001.

Previous studies have shown that germline knockout of *Myh9*, which encodes the non-muscle myosin IIA (NMIIA) heavy chain, is embryonic lethal (48, 49). CD79a-Cre driven knockout of NMIIA results in defects in B-cell development from the pro-B-cell stage onwards (39). Additionally, selective knockout of NMIIA, driven by CD23 expressed in the later developmental stages of B-cells and several other types of immune cells, resulted in reductions in marginal zone and B1b peritoneal B-cells (39). To examine the role of NMIIA in B-cell activation, we generated a B-cell-specific NMIIA knockout by crossing mice expressing the NMIIA heavy-chain (Myh9) floxed allele (Myh9^Flox/Flox^, B6.129S‐*Myh9^tm5Rsad^*/Mmnc) with mice expressing Cre recombinase under the control of the CD19 promotor [B6.129P2(C)-*Cd19^tm1(cre)Cgn^*/J]. RT-PCR analysis showed that NMIIA heavy chain mRNA was reduced ~65% compared to floxed controls but was still present in splenic B-cells, indicating a partial NMIIA conditional knockout (cIIAKO) (**Supplementary Figure 1**). We examined B-cell development in the bone marrow and the periphery in cIIAKO mice using flow cytometry. We did not find any significant changes in the percentages of pre-pro- (A), pro- (B), early pre- (C), late pre- (D), immature (E), and re-circulating mature B-cells (F) in the bone marrow (**Figures 1A, B****)**, and transitional 1 (T1) and 2 (T2), follicular (FO), marginal zone (MZ), and isotype switched (IS) B-cells in the spleen of cIIAKO mice, when compared to floxed littermate control mice (**Figures 1C, D****)**. The total numbers of T1, T2, FO, and IS B-cells per spleen were also comparable between cIIAKO and floxed control mice (**Supplementary Figure 2**).

As mature follicular B-cells can form germinal centers (GCs) after activation, the number of GC B-cells in non-immunized mice reflects the number of spontaneously activated B-cells. We analyzed GC B-cells in the spleen of cIIAKO mice using both flow cytometry and immunohistochemistry. Non-immunized cIIAKO mice had significantly higher percentages of splenic GC B-cells than floxed control mice (**Figures 1E, F****)**. Consistent with this result, immunofluorescence examination showed increased mean fluorescence intensity (MFI) of peanut agglutinin (PNA) staining in the spleen sections of cIIAKO mice, when compared to floxed controls (**Figures 1G, H****)**. Furthermore, the spleen to body weight ratio of cIIAKO mice was significantly higher than that of floxed control mice (**Supplementary Figure 3**). These results indicate that while cIIAKO has no significant impact on B-cell development in the bone marrow and the periphery, it promotes the spontaneous development of GC B-cells. Thus, this cIIAKO mouse model allows us to examine the role of NMIIA in B-cell activation without significant influences from B-cell developmental defects.

### NMIIA Is Recruited to Surface BCRs Upon BCR Activation in a SHIP1-Dependent Manner

**Figure 2:**
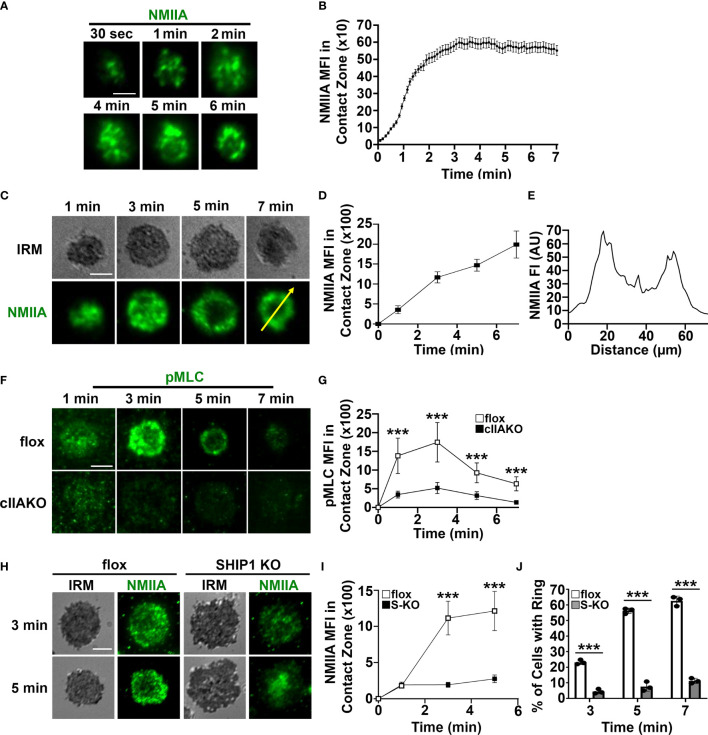
Activated NMIIA is recruited to the B-cell contact zone following BCR activation. B-cells isolated from GFP-NMIIA transgenic mice, WT mice, cIIAKO, SHIP1 KO, and floxed control mice were incubated with Fab’-PLB. **(A)** Representative time-lapse images of GFP-NMIIA B-cells acquired using TIRF. **(B)** The averages (± SD) of GFP-NMIIA MFI in the contact zone of individual B-cells were plotted versus time (>30 cells from 3 mice). **(C)** Activated WT B-cells were fixed at different times, stained for NMIIA, and analyzed using IRM and TIRF. Shown are representative images. **(D)** The averages (± SD) of NMIIA MFI in the B-cell contact zone of individual cells were plotted versus time (>30 cells from 3 mice for each time point). **(E)** The fluorescence intensity (FI) of NMIIA staining across the yellow line in the cell at 7 min in **(C)**. **(F)** Activated floxed control and cIIAKO B-cells were fixed at indicated times and stained for pMLC and analyzed by TIRF. Shown are representative images. **(G)** The averages (± SD) of pMLC MFI in the contact zone of individual B-cells were plotted versus time (>50 cells from 3 mice for each time point). **(H)** Activated floxed control and SHIP1 KO (S-KO) B-cells were fixed at indicated times, stained for NMIIA, and analyzed by IRM and TIRF. Shown are representative images. **(I)** The averages (± SD) of NMIIA MFI in the contact zone of individual cells were plotted versus time. **(J)** The average percentages (± SD) of floxed control and S-KO B-cells exhibiting NMIIA ring-like structures at indicated times (>50 cells from 3 mice for each time point). Scale bar, 5 µm. ***p<0.001.

To determine if NMII is involved in BCR signaling, we first examined whether NMII is recruited to BCR activation sites. We stimulated B-cells using planar lipid bilayers coated with Alexa Fluor 546 labeled, monobiotinylated Fab’ fragment of anti-mouse IgG+M antibody (Fab’-PLB) and visualized NMIIA using B-cells from mice expressing the GFP-NMIIA transgene (**Figures 2A, B****)** or an antibody specific for NMIIA (**Figures 2C–E**). We analyzed NMII recruitment to BCR activation sites using total internal reflection fluorescence (TIRF) microscopy and interference reflection microscopy (IRM). Live-cell TIRF imaging found that GFP-NMIIA was quickly recruited to the plasma membrane regions of primary GFP-NMIIA B-cells contacting Fab’-PLB (contact zone) shortly after cells initiated interactions with Fab’-PLB (**Figures 2A, B****)**. Recruitment of NMIIA to the contact zone was also observed in wt primary B-cells stimulated by Fab’-PLB, after fixation and staining with a NMIIA-specific antibody (**Figures 2C, D****)**, though the recruitment kinetics appeared slower than imaged live cells, likely due to unsynchronized interactions of B-cells with Fab’-PLB. Notably, the recruited NMIIA, visualized with both GFP-NMIIA and NMIIA immunostaining, formed a concentric ring at the outer edge of the B-cell contact zone when B-cells initiated contraction (**Figures 2A, C, E**). Furthermore, the MFI of phosphorylated myosin light chain (pMLC) staining at the contact zone also increased during the first 3 min, indicating that the recruited NMII was activated during this time frame (**Figures 2F, G****)**. There was a significant reduction in the level of pMLC staining in the contact zone of cIIAKO B-cells interacting with Fab’-PLB (**Figures 2F, G****)** and in cIIAKO B-cells activated with F(ab’)_2_ goat anti-mouse IgG+M (**Supplementary Figure 4**), compared to floxed control B-cells. These results suggest that NMIIA is recruited to and activated at BCR activation sites in response to Fab’-PLB and that cIIAKO effectively reduces this recruitment.

We have previously shown that SHIP1 deficiency inhibits B-cell contraction on Fab’-PLB (35), which led to the hypothesis that SHIP1 might be involved in NMII recruitment. To test this hypothesis, we compared NMIIA staining levels at the contact zone of B-cells from B-cell-specific SHIP1 knockout mice (SHIP1 KO or S-KO) to those of floxed littermate controls. Similar to cIIAKO, SHIP1 KO drastically reduced the NMIIA staining level **(** **Figures 2H, I****)** as well as the percentages of B-cells with NMIIA ring-like structures in the contact zone **(** **Figures 2H, J****)**. This result suggests that the inhibitory phosphatase SHIP1 is required for the recruitment of NMIIA to BCR activation sites.

### NMII Is Required for B-Cell Contraction on Ag-Presenting Surfaces

**Figure 3:**
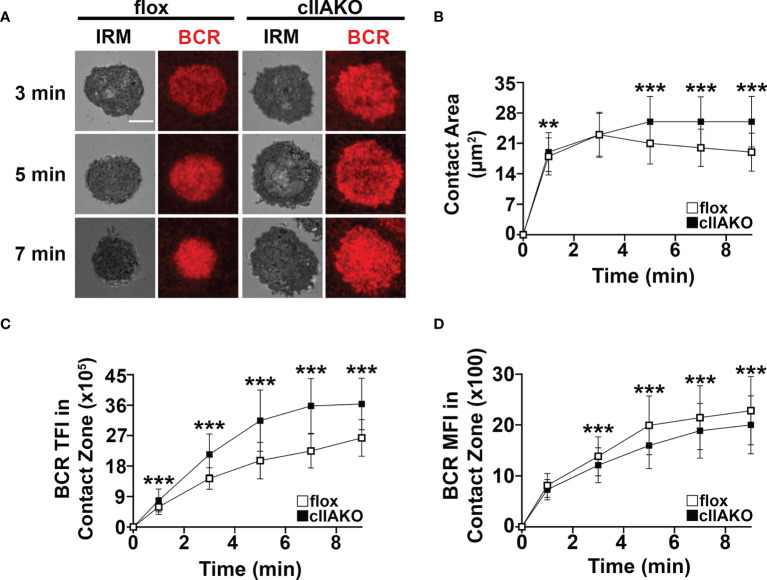
cIIAKO inhibits B-cell contraction and alters BCR clustering in the contact zone. B-cells isolated from floxed control and cIIAKO mice were incubated with Fab’-PLB, fixed at different times, and imaged using IRM and TIRF. Shown are representative IRM and TIRF images of the B-cell contact area and Fab’-engaged BCRs **(A)**. The average contact area (± SD) of individual cells over time was measured using IRM Images **(B)**. The averages (± SD) of Fab’-BCR TFI **(C)** and MFI **(D)** in the contact zone of individual cells over time were measured using TIRF images. >50 cells from 3 mice per time point. Scale bar, 5 µm. **p<0.01, ***p<0.001.

Previous studies have shown that B-cells spread during the first few minutes of interaction with Ag-presenting surfaces, facilitating BCR engagement with Ag and enhancing signaling (33, 34). Following spreading, B-cells undergo contraction, gathering Ag-engaged BCRs into central clusters, promoting BCR signal attenuation (34, 36, 37). To examine whether NMII plays a role in B-cell morphological changes and BCR clustering, we measured the effect of cIIAKO on the area of the B-cell membrane contacting Fab’-PLB over time using IRM and the total fluorescence intensity (TFI) and MFI of Fab’ in the B-cell contact zone (**Figures 3**), as an indicator of BCR clustering (**Supplementary Figure 5****)** (33). We found that the contact area of cIIAKO B-cells on Fab’-PLB increased rapidly, similar to floxed controls in the first 3 min (**Figures 3A, B****)**. After 3 min, the contact area of floxed control B-cells gradually reduced over time, indicating contraction. However, cIIAKO B-cells failed to contract, resulting in significantly larger contact zones than floxed control B-cells (**Figures 3A, B****)**. Consistent with these results, inhibition of NMII motor activity by blebbistatin and inhibition of NMII activation by the Rho-associated protein kinase (ROCK) inhibitor Y27632 reduced the percentage of B-cells exhibiting NMII ring-like structures (**Supplementary Figures 6A, B****)** and delayed B-cell contraction (**Supplementary Figures 6A, C****)**. Concurrent with B-cell spreading on Fab’-PLB, the TFI of Fab’-engaged BCRs in the B-cell contact zone increased over time. The TFI of Fab’ in the contact zone of cIIAKO B-cells was significantly higher than in floxed control B-cells at all time points (**Figure 3C**). However, the MFI of Fab’-engaged BCRs in the contact zone of cIIAKO B-cells was significantly lower than in floxed control B-cells during the contraction phase due to inhibited contraction (**Figure 3D**). While wt and floxed primary B-cells displayed different kinetics and scales of spreading and contraction, likely due to different genetic backgrounds, our data collectively suggest that NMII is required for B-cell contraction, but not B-cell spreading on Ag-presenting surfaces. As the Fab’ MFI in the contact zone has been shown to reflect BCR cluster sizes (35), our results also suggest that NMII is involved in modulating BCR clustering, probably through controlling B-cell contraction.

### NMII Regulates BCR Signaling Induced by Both Surface-Associated and Soluble Stimulation

**Figure 4:**
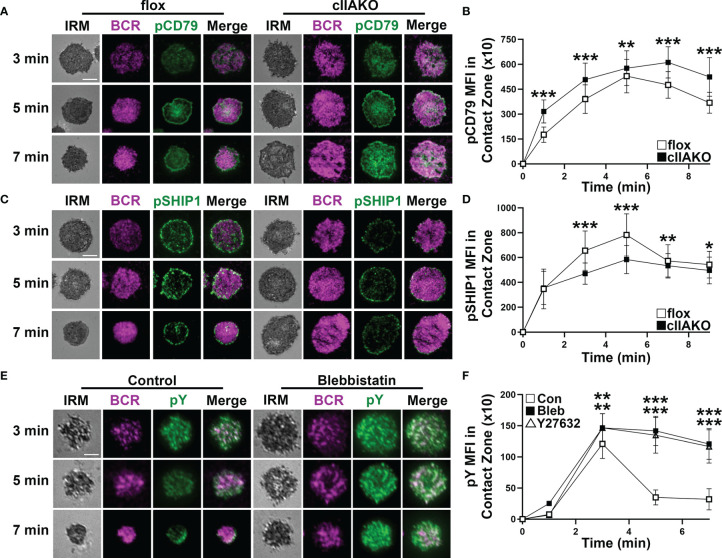
NMIIA inhibition enhances BCR signaling in the B-cell contact zone. **(A–D)** Floxed control and cIIAKO B-cells were incubated with Fab’-PLB for indicated times, fixed, permeabilized, stained for phosphorylated CD79a (pCD79a) **(A, B)** or phosphorylated SHIP1 (pSHIP1) **(C, D)**, and analyzed by IRM and TIRF. Shown are representative IRM and TIRF images of B-cell contact area and pCD79a **(A)** and pSHIP1 staining **(C)** and the averages (± SD) of pCD79a **(B)** or pSHIP1 **(D)** MFI in the contact zone of individual cells measured using TIRF images over time. **(E, F)** WT B-cells untreated or treated with Bleb or Y27632 were incubated with Fab’-PLB, fixed, permeabilized, stained for phosphorylated tyrosine (pY), and analyzed by IRM and TIRF. Shown are representative images **(E)** and the averages (± SD) of pY MFI in the contact zone of individual cells measured using TIRF images over time **(F)**. >50 cells from 3 mice per time point per condition. Scale bar, 5 µm. *p<0.05, **p<0.01, ***p<0.001.

We and others have previously shown that B-cell spreading on Ag-presenting surfaces enhances BCR signaling and that B-cell contraction promotes BCR signaling attenuation (19, 33, 34, 36, 37). This implies a role for NMII in BCR signaling by modulating B-cell morphology. We examined BCR signaling in cIIAKO B-cells and blebbistatin- or Y27632-treated B-cells. B-cells were incubated with Fab’-PLB for varying lengths of time, fixed, permeabilized, and stained for phosphorylated CD79a (Y128, pCD79a, indicating BCR phosphorylation), phosphorylated SHIP1 (Y1020, pSHIP1, representing activation of negative signaling molecules), or phosphor-tyrosine (pY, an indicator of overall signaling). We then imaged the cells with IRM and TIRF and measured the MFI of these signaling molecules in the B-cell contact zone. We found that the MFI of pCD79a in the contact zone of cIIAKO B-cells during both the activation and attenuation stages was higher than in floxed control B-cells (**Figures 4A, B****)**. In contrast, the MFI of pSHIP1 in the contact zone of cIIAKO B-cells was significantly lower than in floxed control B-cells (**Figures 4C, D****)**. Blebbistatin- or Y27632-treated wt primary B-cells showed sustained elevated MFI of pY in the contact zone at 5 and 7 min, while the pY MFI of vehicle control B-cells decreased significantly after 3 min (**Figures 4E, F****)**. These data suggest a negative regulatory role for NMII in B-cell signaling triggered by surface-associated Ag.

**Figure 5:**
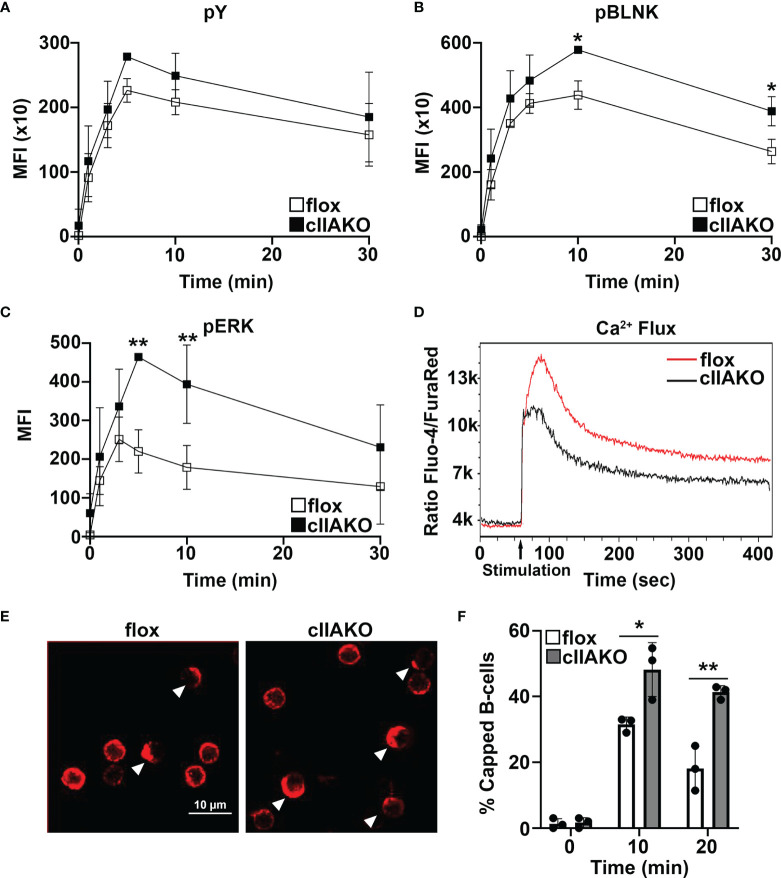
BCR signaling and BCR capping are enhanced in cIIAKO B-cells in response to soluble stimulation. **(A–C)** B-cells from floxed control and cIIAKO mice were activated with F(ab’)_2_ goat anti-mouse IgG+M for indicated times, fixed, permeabilized, labeled for pY **(A)**, pBLNK **(B)**, and pErk **(C)**, and analyzed by flow cytometry. Shown are the average MFI (± SD) of three independent experiments. **(D)** Representative histograms of Ca^2+^ flux in floxed control and cIIAKO B-cells activated with F(ab’)_2_ goat anti-mouse IgG+M by flow cytometry. **(E, F)** Representative confocal images of activated floxed control and cIIAKO B-cells showing BCR capping at 10 min **(E)** and the average percentages (± SD) of B-cells with BCR capping at indicated times **(F)**. Scale bar, 10 µm. n=3. *p< 0.05, **p<0.01.

To determine if NMII also plays a role in BCR signaling activated by soluble Ag, we compared BCR signaling in cIIAKO and floxed control B-cells triggered by BCR cross-linking with F(ab’)_2_ anti-mouse IgG+M using flow cytometry. Compared to floxed control B-cells, cIIAKO B-cells showed slightly increased levels of pY (**Figure 5A**), significantly elevated levels of phosphorylated BLNK (Y84, pBLNK), a BCR proximal signaling scaffolding protein **(** **Figure 5B****)**, and phosphorylated ERK (T202/Y204, pERK), a key component in the MAPK signaling pathway (**Figure 5C**), but no changes in their protein levels (**Supplementary Figure 7**). The increases in the levels of these phosphorylated signaling molecules were primarily detected during the signaling attenuation stage (**Figures 5A–C** and **Supplementary Figure 8**). However, the Ca^2+^ signaling level in cIIAKO B-cells, measured by the ratio of Fluo-4 to Fura Red using flow cytometry, was lower than in floxed control cells (**Figure 5D**). To further examine the impact of cIIAKO on surface BCR clustering induced by soluble Ag stimulation, we measured the percentage of B-cells with surface BCRs polarized to one pole of a cell, called “capping” (**Figure 5E**). We found higher percentages of cIIAKO B-cells with BCR caps than floxed control B-cells at 10 and 20 min (**Figures 5E, F****)**. These data suggest that NMIIA also regulates BCR signaling triggered by soluble Ag, primarily promoting signaling attenuation.

### NMIIA Facilitates Antibody Affinity Maturation and Reduces Autoantibody Production

**Figure 6:**
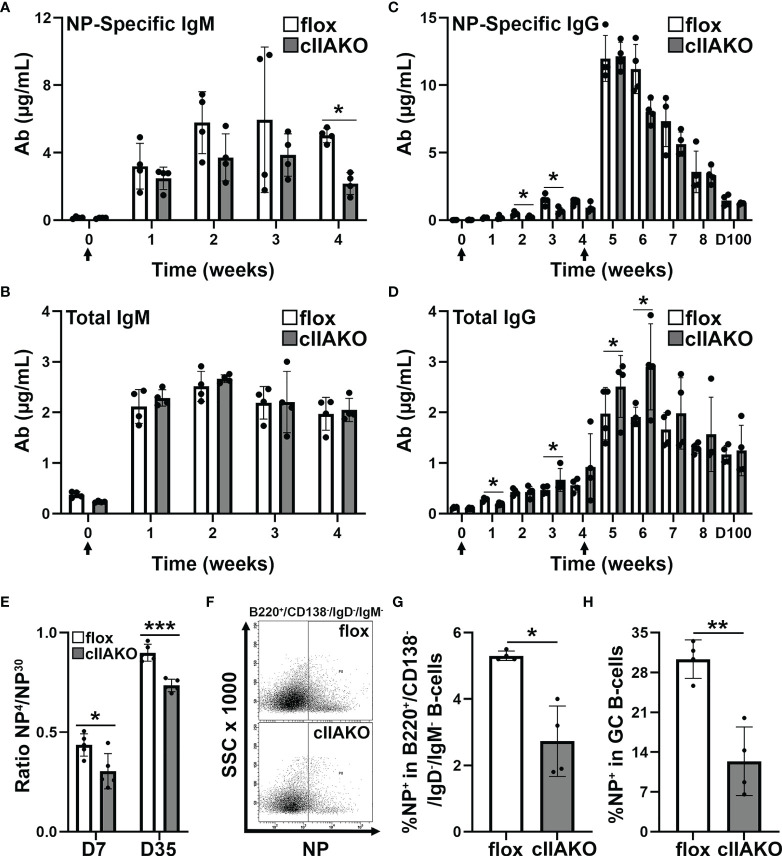
B-cell affinity maturation is impaired in cIIAKO mice. **(A–D)** 6~8 weeks old floxed control and cIIAKO mice (n=4) were immunized with NP-KLH in Sigma Adjuvant System on day 0 and boosted on day 28. NP-specific IgM **(A)**, total IgM **(B)**, NP-specific IgG **(C)**, and total IgG **(D)** in the serum were determined by ELISA (mean µg/ml, ± SD). **(E)** Relative affinity of NP-specific IgG 7 and 35 days post NP-KLH immunization was assessed as ratios of IgG bound to NP_4_- versus NP_30_-BSA by ELISA (± SD, n=4~5). **(F–H)** Splenic cells from floxed control and cIIAKO mice 72 days post second immunization were stained for NP binding, B220, CD138, IgM, and IgD, or CD95 and GL-7, and analyzed by flow cytometry **(F)**. Percentages of NP^+^/B220^+^/CD138^-^/IgM^-^/IgG^-^ cells **(G)** and NP^+^/B220^+^/CD95^+^/GL-7^+^ B-cells **(H)** were quantified as Ag-specific isotype switched and GC B-cells, respectively. Data points represent individual mice. n=4. *p<0.05, **p<0.01, ***p<0.001.

We next examined how the regulatory role of NMII in BCR signaling contributed to BCR-initiated antibody responses. We immunized mice with the T-dependent Ag 4-hydroxy-3-nitrophenylacetyl hapten conjugated to keyhole limpet hemocyanin (KLH) twice, 28 days apart, and quantified NP-specific and total IgM and IgG in the serum using ELISA. In response to NP-KLH immunization, NP-specific IgM and total IgM levels in cIIAKO mice were similar to levels detected in floxed control mice, except for a reduction in Ag-specific IgM at week 4 (**Figures 6A, B****)**. Similarly, serum levels of NP-specific IgG were not significantly affected in cIIAKO mice, when compared to floxed control mice (**Figure 6C**). However, the level of total IgG was significantly elevated in cIIAKO mice after both the first and second immunizations (**Figure 6D**). We further evaluated antibody affinity in NP-KLH immunized mice by determining the ratio of IgG bound to low-valent (NP_4_) and high-valent (NP_30_) Ag by ELISA, which reflected the fraction of total NP-binding IgG (binding to NP_30_) with relatively high affinity (binding to NP_4_). While the fraction of high-affinity NP-specific IgG in cIIAKO mice increased 7 days after the second immunization (D35), compared to 7 days after the first immunization (D7), the high-affinity fractions were significantly lower in cIIAKO mice than in floxed control mice after both the first and second immunizations (**Figure 6E**). We quantified the percentages of NP-specific B-cells among isotype switched B-cells (B220^+^IgD^-^IgM^-^) and germinal center B-cells (B220^+^GL7^+^CD95^+^) in the spleen, 72 days after the second immunization, using NP-APC staining and flow cytometry. The spleens of cIIAKO mice had decreased percentages of both NP-specific isotype-switched B-cells (**Figures 6F, G****)** and germinal center B-cells (**Figure 6H**). These data suggest that cIIAKO mice mount T-dependent antibody responses with impaired affinity maturation.

**Figure 7:**
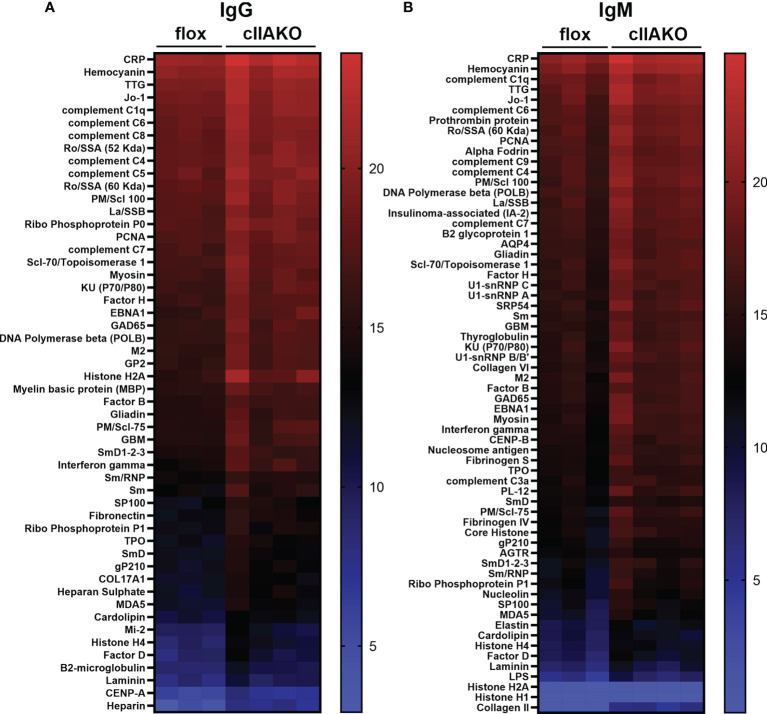
Autoantibody production was elevated in cIIAKO mice compared to floxed control mice. Autoreactive IgG **(A)** and IgM **(B)** in the sera of unimmunized 12 months old floxed control and cIIAKO mice (n=3-4) were screened using autoantigen arrays. Autoantibodies were detected with fluorescent antibodies for either mouse IgG or IgM. The net fluorescent intensity (NFI) and signal-to-noise ratio (SNR) were calculated for each autoantigen. NFI and SNR were used to calculate Ab scores, then filtered antibody scores (whose percent of SNR>3 across all samples is less than 10) were used for analysis. Shown are heatmaps, where each row represents an autoantigen and each column represents a serum sample from a mouse. The red color represents the highest filtered Ab score, while the blue represents the lowest filtered Ab-scores. Filtered Ab scores were sorted from largest to smallest mean filtered Ab score. Only autoantibodies with statistically significant different filtered Ab-scores between cIIAKO and floxed control mice are shown. *p ≤* 0.05.

The elevated serum IgG levels and the reduced NP-specific germinal center B-cells suggested increased levels of non-specific antibodies, possibly autoantibodies, in cIIAKO mice. To test this hypothesis, we screened autoantibody levels in the sera of 12 month-old cIIAKO and floxed control mice using Autoantigen Microarray analysis (Genomic and Microarray Core Facility of the University of Texas Southwestern Medical Center). We found significant increases in the levels of both serum IgG (**Figure 7A**) and IgM (**Figure 7B**) bound to a large number of autoantigens in cIIAKO mice, compared to floxed control mice. Many of the identified autoantigens are commonly associated with systemic autoimmune diseases, such as systemic lupus erythematosus, including antibodies specific for nuclear proteins, C-reactive protein, hemocyanin, complement C1q, and β2-microglobulin (50, 51), in cIIAKO mice, compared to floxed control mice. This result suggests that NMII is involved in controlling the development of non-specific or autoreactive B-cells.

## Discussion

This study took advantage of a partial NMIIA gene deletion mouse model with limited effects on B-cell development to examine the B-cell intrinsic function of NMIIA in BCR signaling and BCR-initiated antibody responses. Inhibition of NMII by cIIAKO or pharmacological inhibitors inhibits B-cell contraction on Ag-presenting surfaces and enhances BCR capping in response to soluble stimulation, which leads to prolonged BCR signaling. NMII is recruited to BCR activation sites in a SHIP1-dependent manner and forms a peripheral ring at the outer edge of the B-cell membrane region in contact with Ag-presenting surfaces, facilitating B-cell contraction. Prolonged BCR signaling in cIIAKO mice is associated with reductions in Ag-specific GC B-cells, isotype-switched B-cells, and high-affinity IgG in response to immunization with T-dependent Ag. The elevated signaling also leads to increases in autoantibodies. Collectively, our results suggest an essential role for NMII in the negative regulation of BCR signaling. NMII negatively regulates BCR signaling likely by driving B-cell contraction on Ag-presenting surfaces and inhibiting BCR capping induced by soluble stimulation. The NMII-facilitated BCR signaling attenuation is important for controlling the expansion and differentiation of non-specific B-cells.

The roles of NMII in cytokinesis and cell migration have been extensively studied (38). Mutations in NMII genes are associated with various diseases (52, 53). In addition to those ubiquitous functions, NMII, and particularly NMIIA, the dominant isoform of NMII expressed in B-cells (39), has been implicated in B-cell signaling. B-cells from CD23-Cre-driven NMIIA-deficient mice upregulate the expression of activation markers, even though they mount poor antibody responses to immunization (39). Here, we identify the role of NMII in BCR signaling and further reveal potential underlying mechanisms. Our studies show that NMII facilitates BCR signaling attenuation, and that this negative regulation of BCR signaling is in response to both surface-associated and soluble Ag. In B-cells activated with Fab’-PLB, NMII promotes BCR signaling attenuation by driving B-cell contraction after spreading. We have previously shown that delaying B-cell contraction by B-cell-specific knockout of N-WASp, an actin nucleation promoting factor, or through germline knockout of Abp1, slows the centripetal movement of BCR microclusters while enhancing and prolonging BCR signaling (36, 37). Thus, NMII likely plays a critical role in F-actin reorganization, driving B-cell contraction. Our results also indicate that NMIIA negatively regulates BCR signaling induced by soluble stimulation, by limiting BCR cap formation. B-cell binding of soluble Ag capable of crosslinking surface BCRs leads to receptor clustering and subsequent accumulation of BCR microclusters at one pole of the cell, forming a BCR cap. BCR caps function as supramolecular activation complexes (17, 54). We have previously shown that the actin cytoskeleton is required for BCR cap formation (33). Together, these data suggest that NMII negatively regulates BCR signaling using distinctive mechanisms based on the nature of the Ag, surface-associated or soluble. How NMII works with the actin cytoskeleton to inhibit BCR signaling induced by different Ag types is a question that remains to be answered.

NMII may negatively regulate BCR signaling by facilitating BCR endocytosis. NMII is critical for B-cells to pull Ag from Ag-presenting surfaces for endocytosis (40, 42, 43, 55). BCR-mediated Ag endocytosis initiates Ag processing and presentation and also removes BCRs from surface signalosomes, potentially attenuating BCR proximal signaling. However, NMII is not required for BCR endocytosis of soluble Ag (40, 42). Additionally, B-cell endocytosis of surface-associated Ag occurs much later than BCR proximal signaling (40, 42, 43). These findings argue against NMII-dependent BCR endocytosis as a possible mechanism for BCR signaling attenuation.

Our signaling analysis further shows that while the levels of phosphorylated BLNK and Erk are higher in cIIAKO than floxed control B-cells at 5 and/or 10 min in response to soluble stimulation, Ca^2+^ influx, occurring within seconds, was lower in cIIAKO than floxed control B-cells. This result suggests that NMII may be involved in regulating the activation of BCR signaling. Future studies should analyze the effects of cIIAKO or NMII inhibition on signaling pathways upstream of Ca^2+^ influx, including PI3K and PLCγ (2, 56). Additionally, NMII may facilitate Ca^2+^ influx by triggering mechanosensing ion channels, like Piezo1 (57, 58), as NMII generates traction forces at surface BCRs (40, 59). These hypotheses remain to be tested.

In contrast to CD23-Cre-driven B-cell-specific NMIIA knockout (39), our partial cIIAKO did not dramatically affect IgM and IgG antibody responses to immunization with NP-KLH, a T-dependent Ag. However, we detected a significant increase in the total levels of serum IgG. This elevated serum IgG level was concurrent with decreases in Ag-specific germinal center B-cells and isotype switched B-cells and the fraction of high-affinity IgG. These results indicate that this B-cell-specific NMIIA-deficiency causes increases in low-affinity and non-specific B-cells, potentially leading to a weakened antibody response to immunization and elevated autoantibody levels. Indeed, our autoantibody screen confirmed increased autoantibody levels in cIIAKO mice compared to floxed control mice. Notably, many elevated autoantibodies are associated with systemic autoimmune diseases, such as systemic lupus erythematosus (50, 51). These findings strongly support a relationship between NMII-mediated negative regulation of BCR signaling and negative selection of non-specific and autoreactive B-cells. The selection of high-affinity B-cells in germinal centers is heavily dependent on the capability of B-cells to compete for Ag engagement and capture through the BCR (9), which enables B-cells to induce signaling and acquire T-cell help. Low-affinity and non-specific B-cells eventually die through apoptosis, due to a lack of BCR signaling and T-cell help (60, 61). NMII-mediated negative regulation of BCR signaling may contribute to the death of non-specific and autoreactive B-cells by limiting the necessary survival signals from the BCR (62). NMII is also required for B-cells to internalize Ag in an affinity-dependent manner from Ag-presenting cells (39, 40), such as follicular dendritic cells in germinal centers, for presentation to T-cells, a critical step for affinity maturation. NMII may also contribute to B-cell affinity maturation by modulating B-cell migration and cytokinesis.

This study shows that BCR activation by Fab’-PLB induces rapid recruitment of NMII to BCR activation sites, consistent with previous reports (40, 43). Phosphorylated MLC is also recruited to the B-cell contact zone, suggesting that the recruited NMII is activated by BCR signaling. The mechanism by which NMII is activated is well-studied (38, 63). The motor activity of NMII is controlled by its regulatory light chain MLC and is activated by MLC phosphorylation through MLC kinase (MLCK) and Rho-associated kinase (ROCK) (64). MLCK is activated by Ca^2+^-calmodulin, while ROCK is activated through Rho-GTPases (64, 65). Our findings that both the NMII motor inhibitor blebbistatin and the ROCK inhibitor Y27632 delay B-cell contraction and BCR signaling attenuation suggest that BCR-induced activation of NMII involves ROCK. We further show that both NMII and pMLC accumulate at the outer edge of the contact zone, forming a ring-like structure, during B-cell contraction. Inhibiting NMII motor activity with blebbistatin and NMII activation by Y27632 reduces the percentage of B-cells exhibiting the NMII ring-like structure and delays B-cell contraction. These results suggest that NMII plays a direct role in B-cell membrane contraction. However, B-cell contraction appears to be less impacted in inhibitor-treated cells when compared to cIIAKO B-cells. The differences observed may be attributed to differential mouse backgrounds or the minimal effective inhibitor concentrations and treatment durations used to minimize potential off-target effects.

Surprisingly, we found that the recruitment of NMII to the B-cell contact zone depends on SHIP1, a phosphoinositol-5 phosphatase, using a B-cell-specific SHIP1 knockout mouse model (35, 66). SHIP1 is known to be critical for BCR signaling attenuation by removing PI(4,5)P_2_ and PI(3,4,5)P_3_, membrane docking sites for signaling molecules, such as PLCγ2, Akt, and Btk (24, 25, 67, 68), which are upstream of NMII activation pathways. These studies suggest a role for SHIP1 in inhibiting rather than promoting NMII activation, contradictory to our findings. Our previously published results showed that SHIP1 KO, like cIIAKO, inhibits B-cell contraction (35), supporting a role for SHIP1 in facilitating NMII recruitment to promote B-cell contraction. Additionally, our previous studies show that SHIP1 KO drastically changes the actin organization in the B-cell contact zone, increasing F-actin accumulation and inhibiting actin-driven BCR centripetal movement by enhancing Btk-mediated WASp activation (35). Together, these data support the notion that NMII recruitment and ring-like structure formation require a unique actin organization at the B-cell contact zone in addition to signaling activation.

This study reveals a critical role for NMII in BCR signaling attenuation. The data accumulated thus far enable us to propose a working model for this novel role of NMII in B-cells. Upon BCR engagement by surface-associated Ag, NMII is activated through BCR signaling and recruited to the B-cell contact zone. NMII recruitment is regulated by actin organization, which is in turn controlled by a signaling balance between stimulatory kinase Btk and inhibitory phosphatase SHIP1. NMII redistributes from BCR clusters to the outer edge of the B-cell contact zone along with the actin cytoskeleton during the late stages of B-cell spreading. Actomyosin-generated traction forces then contract the B-cell plasma membrane, driving the merge of BCR clusters and subsequent attenuation of BCR signaling. Distinctively, NMII promotes signaling attenuation in B-cells activated by soluble Ag by limiting the formation of BCR caps (supramolecular activation complexes). While mechanistic details underlying NMII’s function in different B-cell subsets under different activation conditions remains to be explored, our data point to a critical role of NMII in controlling BCR signaling and autoreactive B-cells.

## Data Availability Statement

The original contributions presented in the study are included in the article/**Supplementary Material**. Further inquiries can be directed to the corresponding author.

## Ethics Statement

The animal study was reviewed and approved by University of Maryland College Park Institutional Animal Care and Use Committee.

## Author Contributions

MS-F, ML, and CL designed and performed the experiments, analyzed the data, and prepared figures. MS-F wrote the manuscript. AU provided materials and input on the manuscript. WS designed and supervised the research and wrote the manuscript. All authors contributed to the article and approved the submitted version.

## Funding

This work was supported by NIH grants AI122205 and GM064625.

## Conflict of Interest

The authors declare that the research was conducted in the absence of any commercial or financial relationships that could be construed as a potential conflict of interest.

## Publisher’s Note

All claims expressed in this article are solely those of the authors and do not necessarily represent those of their affiliated organizations, or those of the publisher, the editors and the reviewers. Any product that may be evaluated in this article, or claim that may be made by its manufacturer, is not guaranteed or endorsed by the publisher.

## Supplementary Material

Supplementary Figure 1 Partial knockout of NMIIA. **(A)** Shown is a representative gel image of RT-PCR products of the 130 base sequence within the deleted exon 3 of the *MHY9* gene. RNA was isolated from floxed control and cIIAKO B-cells.** (B)** The relative intensity of bands from the PCR products of cIIAKO and floxed control mice was determined using NIH ImageJ. n=3. **p*<0.05.

Supplementary Figure 2 CD19-Cre-driven NMIIA knockout (cIIAKO) does not impact B-cell development. Cells from the spleens of floxed control and cIIAKO mice were labeled for surface markers of transitional 1 (T1), transitional 2 (T2), follicular (FO), marginal zone (MZ), and isotype switched (IS) B-cells, and analyzed by flow cytometry. Shown are the average number (± SD) of each B-cell subset per mouse. Data points represent individual mice. n=3. **p*<0.05

Supplementary Figure 3 Ratios of mouse spleen to body weight. The average (± SD) of spleen weight as a ratio of total body mass from floxed control and cIIAKO mice. Data points represent individual mice. n=8, 6-8 weeks old. **p*<0.05. ​

Supplementary Figure 4 The level of phosphorylated non-muscle myosin II light chain (pMLC) is reduced in cIIAKO B-cells compared to floxed control B-cells. Floxed control and cIIAKO B-cells were activated with F(ab’)_2_ goat anti-mouse IgG+M for 10 min, fixed, permeabilized, stained for pMLC, and analyzed by flow cytometry. Shown is a representative histogram of three independent experiments.

Supplementary Figure 5 Fab’- but not transferrin (Tf)-PLB induces BCR clustering. B-cells from floxed control mice were incubated with Fab’-PLB or Tf-PLB for 3 min and analyzed by IRM and TIRF. Shown are representative IRM and TIRF images of the B-cell contact area and Fab’ accumulation in the contact area. Scale bar, 5 µm.

Supplementary Figure 6 Inhibition of NMII inhibits the formation of NMIIA ring-like structures and B-cell contraction. B-cells isolated from WT mice were pre-treated with vehicle control (Con), blebbistatin (Bleb), or Y27632 and incubated with Fab’-PLB in the presence or absence of the inhibitor. Activated B-cells were fixed at indicated times, stained for NMIIA, and imaged using IRM and TIRF. Shown are representative IRM and TIRF images of the B-cell contact zone **(A),** the average percentage (± SD) of B-cells exhibiting NMIIA ring-like structures using TIRF images **(B),** and the average contact area (± SD) of individual cells over time measured using IRM Images **(C)**. >50 cells per time point per condition from three mice. Scale bar, 5 µm. ***p*<0.01, ****p*<0.001.

Supplementary Figure 7 Protein expression levels of signaling molecules in cIIAKO and floxed control B-cells. Floxed control and cIIAKO B-cells were fixed, permeabilized, labeled for CD79 **(A)**, BLNK **(B)**, Erk **(C)**, and SHIP1 **(D)**, and analyzed by flow cytometry. Shown are representative histograms from 3 independent experiments. ​

Supplementary Figure 8 BCR signaling in cIIAKO B-cells is enhanced in response to soluble stimulation. B-cells from floxed control and cIIAKO mice were activated with F(ab’)_2_ goat anti-mouse IgG+M, fixed, permeabilized, labeled for pY **(A)**, pBLNK **(B),** and pErk **(C)**, and analyzed by flow cytometry. Shown are representative histograms of pY **(A)** and pERK **(C)** at 5 min and pBLNK at 10 min **(B)** from three independent experiments. ​
