# Supplementary figures and images for: Non-Muscle Myosin II Is Essential for the Negative Regulation of B-Cell Receptor Signaling and B-Cell Activation
Source: Front Immunol. 2022 Apr 14;13:842605. doi: 10.3389/fimmu.2022.842605 (PMC9047714; doi:10.3389/fimmu.2022.842605)

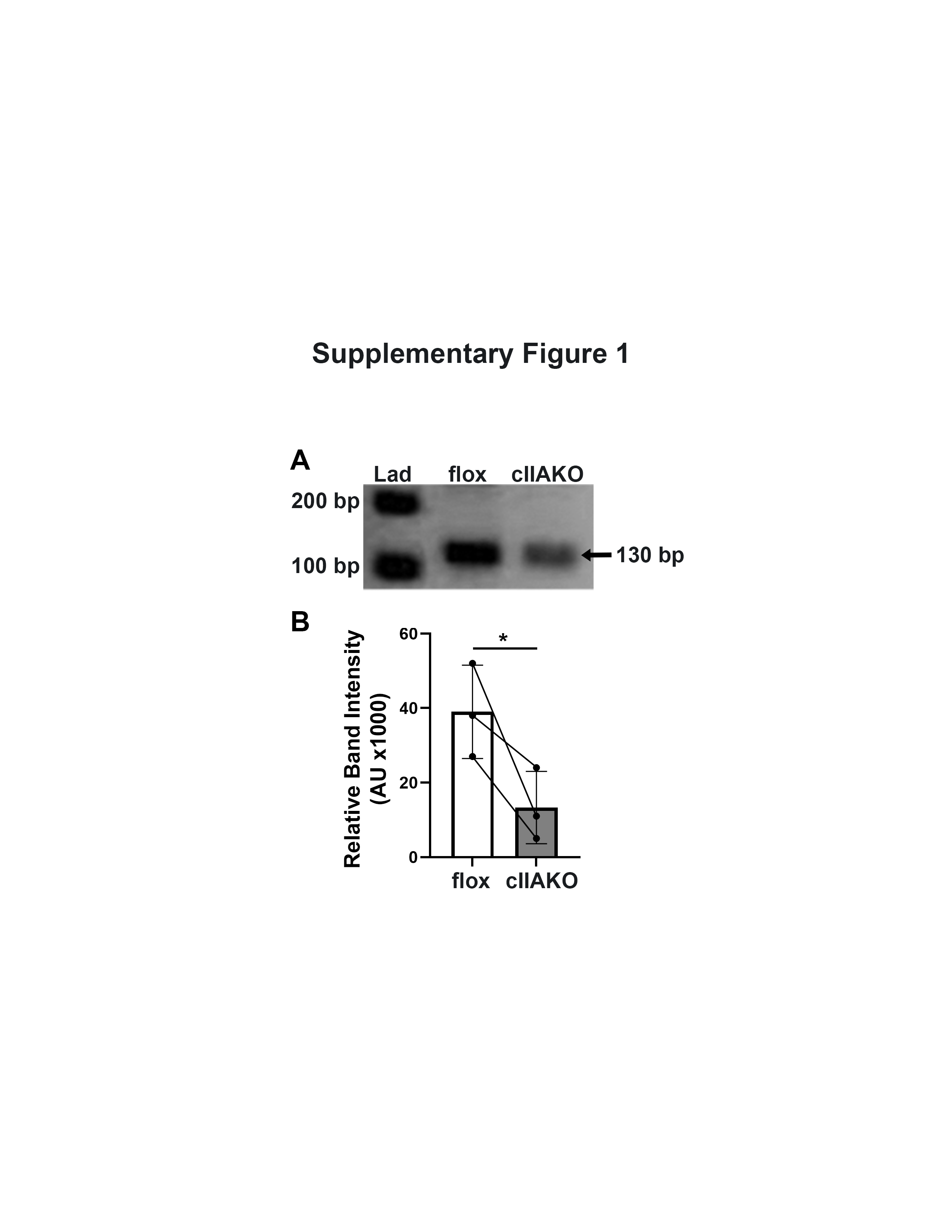

Supplement: Supplementary Figure 1 — Partial knockout of NMIIA. (A) Shown is a representative gel image of RT-PCR products of the 130 base sequence within the deleted exon 3 of the MHY9 gene. RNA was isolated from floxed control and cIIAKO B-cells. (B) The relative intensity of bands from the PCR products of cIIAKO and floxed control mice was determined using NIH ImageJ. n=3. *p<0.05. [file Image_1.tif]

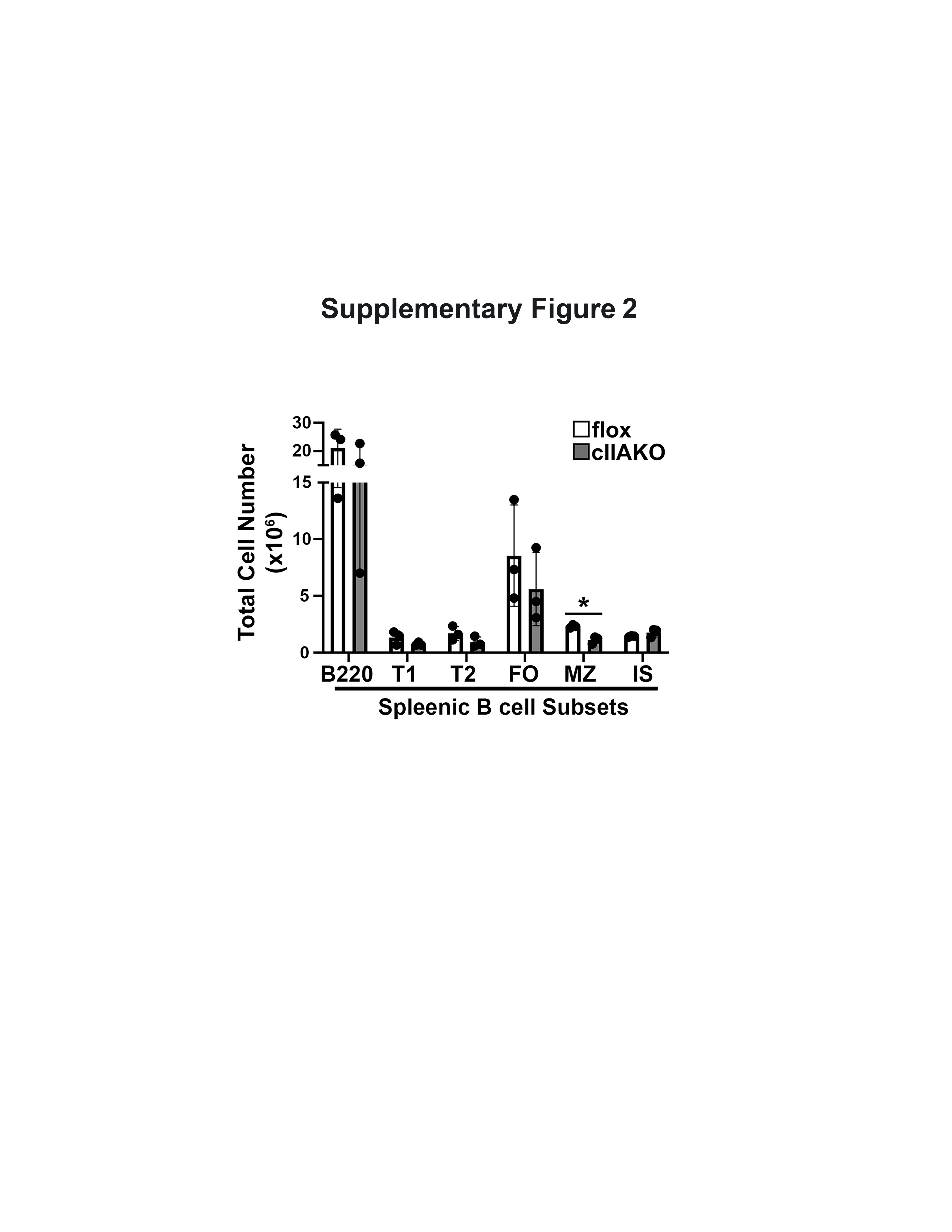

Supplement: Supplementary Figure 2 — CD19-Cre-driven NMIIA knockout (cIIAKO) does not impact B-cell development. Cells from the spleens of floxed control and cIIAKO mice were labeled for surface markers of transitional 1 (T1), transitional 2 (T2), follicular (FO), marginal zone (MZ), and isotype switched (IS) B-cells, and analyzed by flow cytometry. Shown are the average number (± SD) of each B-cell subset per mouse. Data points represent individual mice. n=3. *p<0.05 [file Image_2.tif]

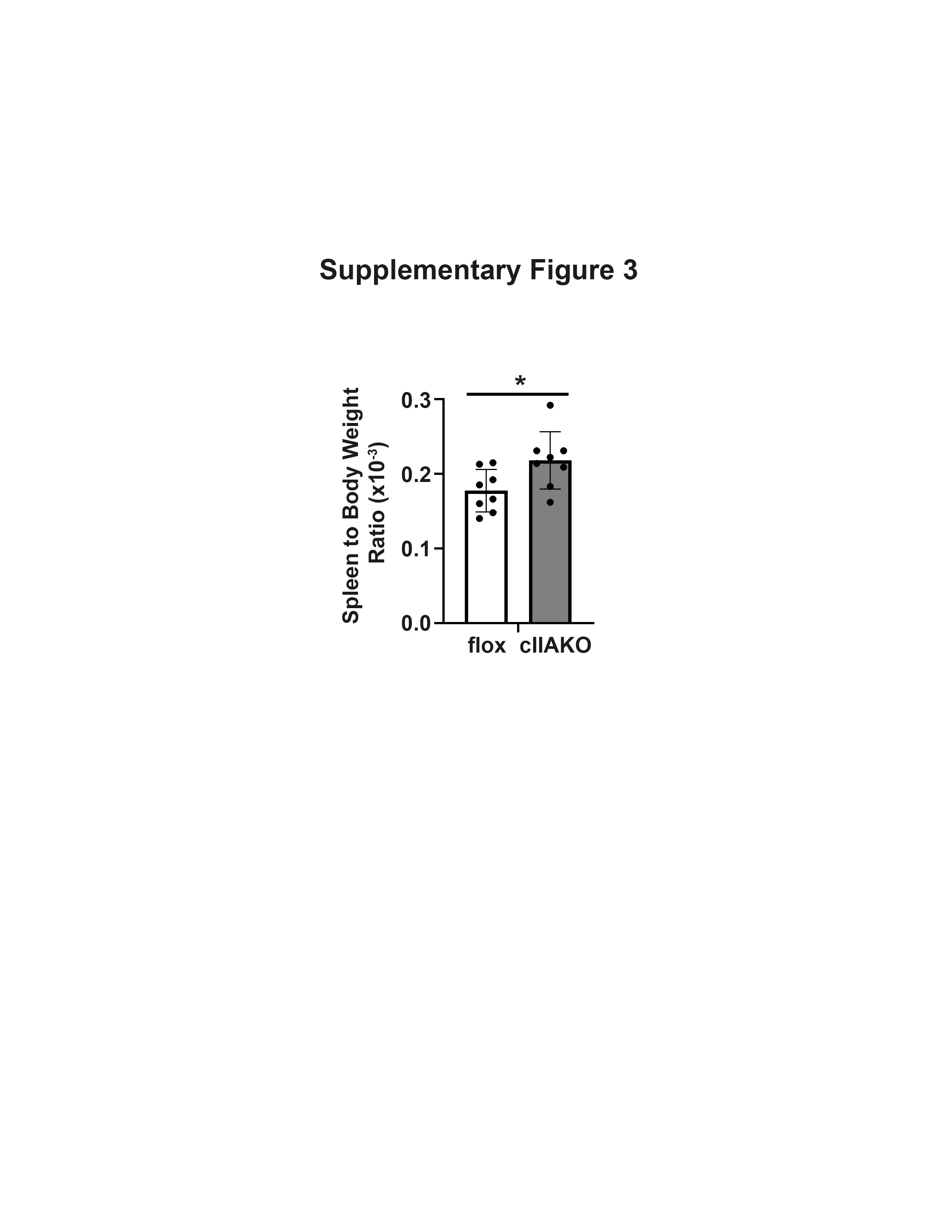

Supplement: Supplementary Figure 3 — Ratios of mouse spleen to body weight. The average (± SD) of spleen weight as a ratio of total body mass from floxed control and cIIAKO mice. Data points represent individual mice. n=8, 6-8 weeks old. *p<0.05. ​ [file Image_3.tif]

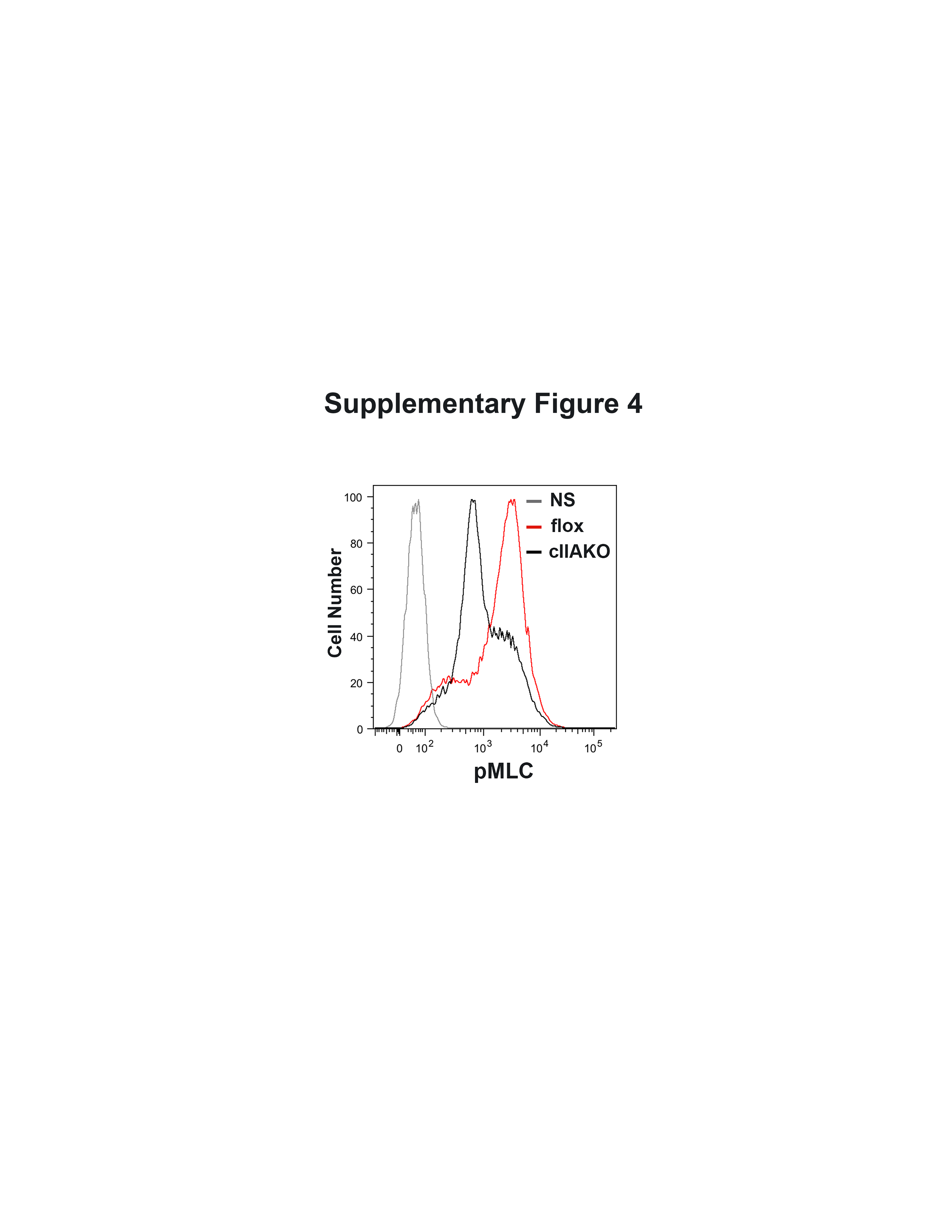

Supplement: Supplementary Figure 4 — The level of phosphorylated non-muscle myosin II light chain (pMLC) is reduced in cIIAKO B-cells compared to floxed control B-cells. Floxed control and cIIAKO B-cells were activated with F(ab’)2 goat anti-mouse IgG+M for 10 min, fixed, permeabilized, stained for pMLC, and analyzed by flow cytometry. Shown is a representative histogram of three independent experiments. [file Image_4.tif]

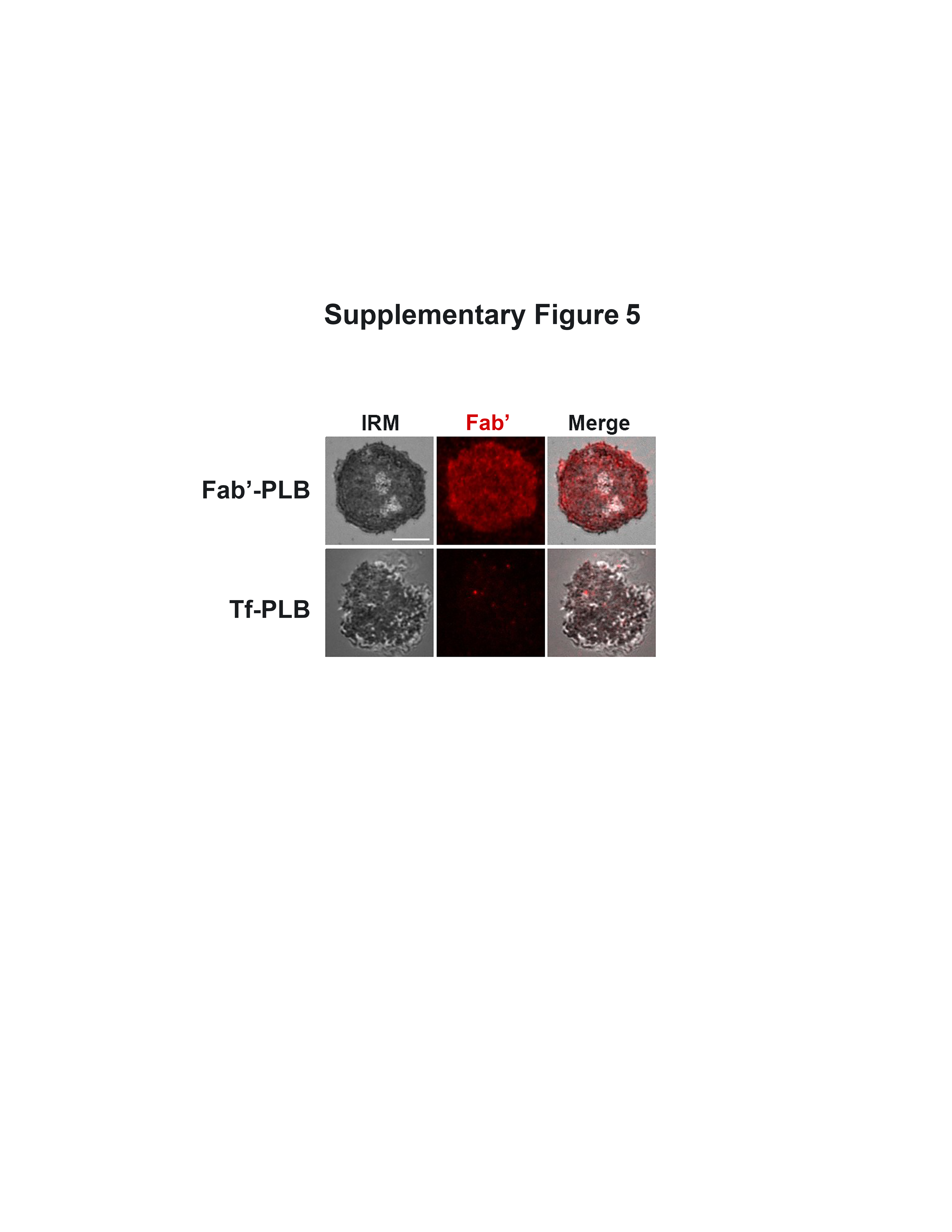

Supplement: Supplementary Figure 5 — Fab’- but not transferrin (Tf)-PLB induces BCR clustering. B-cells from floxed control mice were incubated with Fab’-PLB or Tf-PLB for 3 min and analyzed by IRM and TIRF. Shown are representative IRM and TIRF images of the B-cell contact area and Fab’ accumulation in the contact area. Scale bar, 5 µm. [file Image_5.tif]

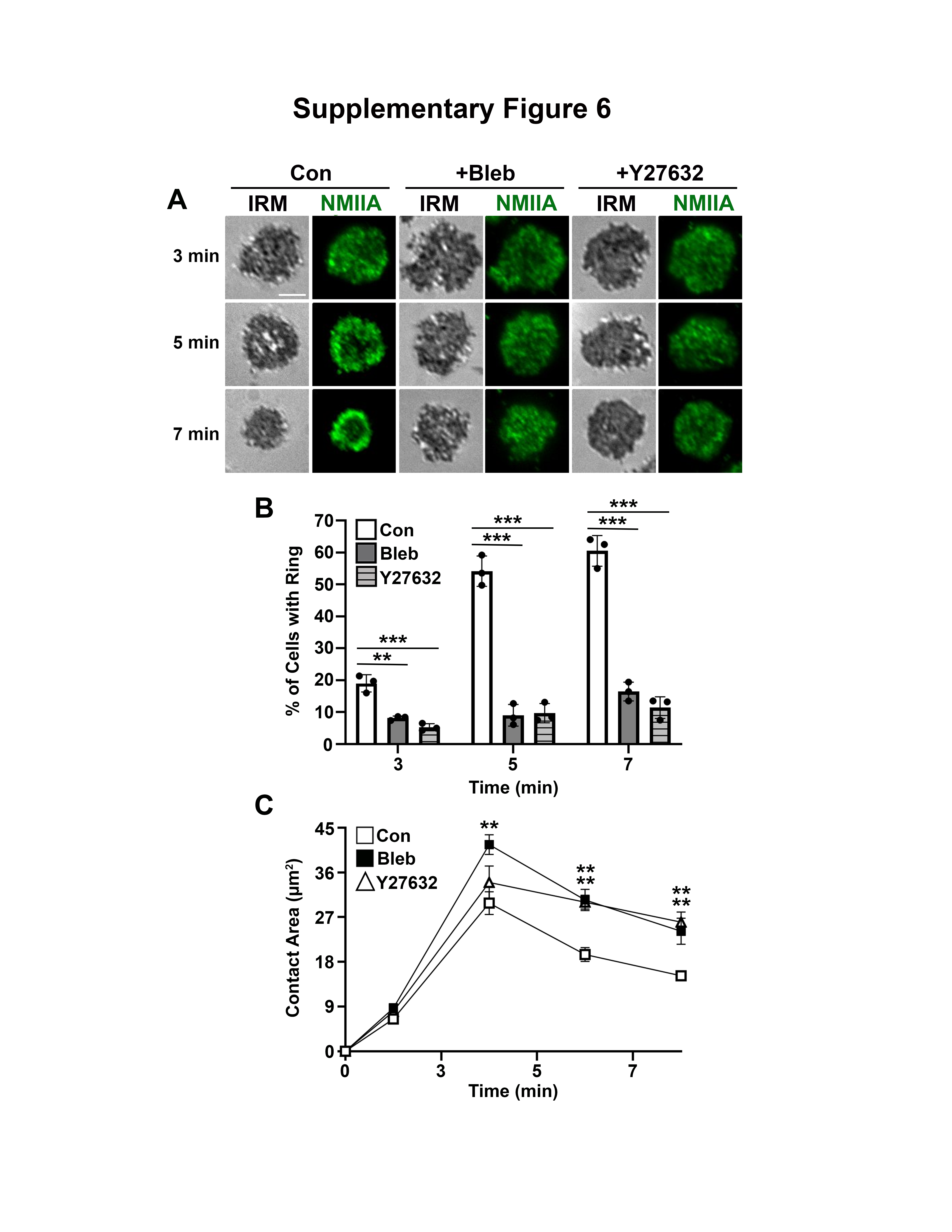

Supplement: Supplementary Figure 6 — Inhibition of NMII inhibits the formation of NMIIA ring-like structures and B-cell contraction. B-cells isolated from WT mice were pre-treated with vehicle control (Con), blebbistatin (Bleb), or Y27632 and incubated with Fab’-PLB in the presence or absence of the inhibitor. Activated B-cells were fixed at indicated times, stained for NMIIA, and imaged using IRM and TIRF. Shown are representative IRM and TIRF images of the B-cell contact zone (A), the average percentage (± SD) of B-cells exhibiting NMIIA ring-like structures using TIRF images (B), and the average contact area (± SD) of individual cells over time measured using IRM Images (C). >50 cells per time point per condition from three mice. Scale bar, 5 µm. **p<0.01, ***p<0.001. [file Image_6.tif]

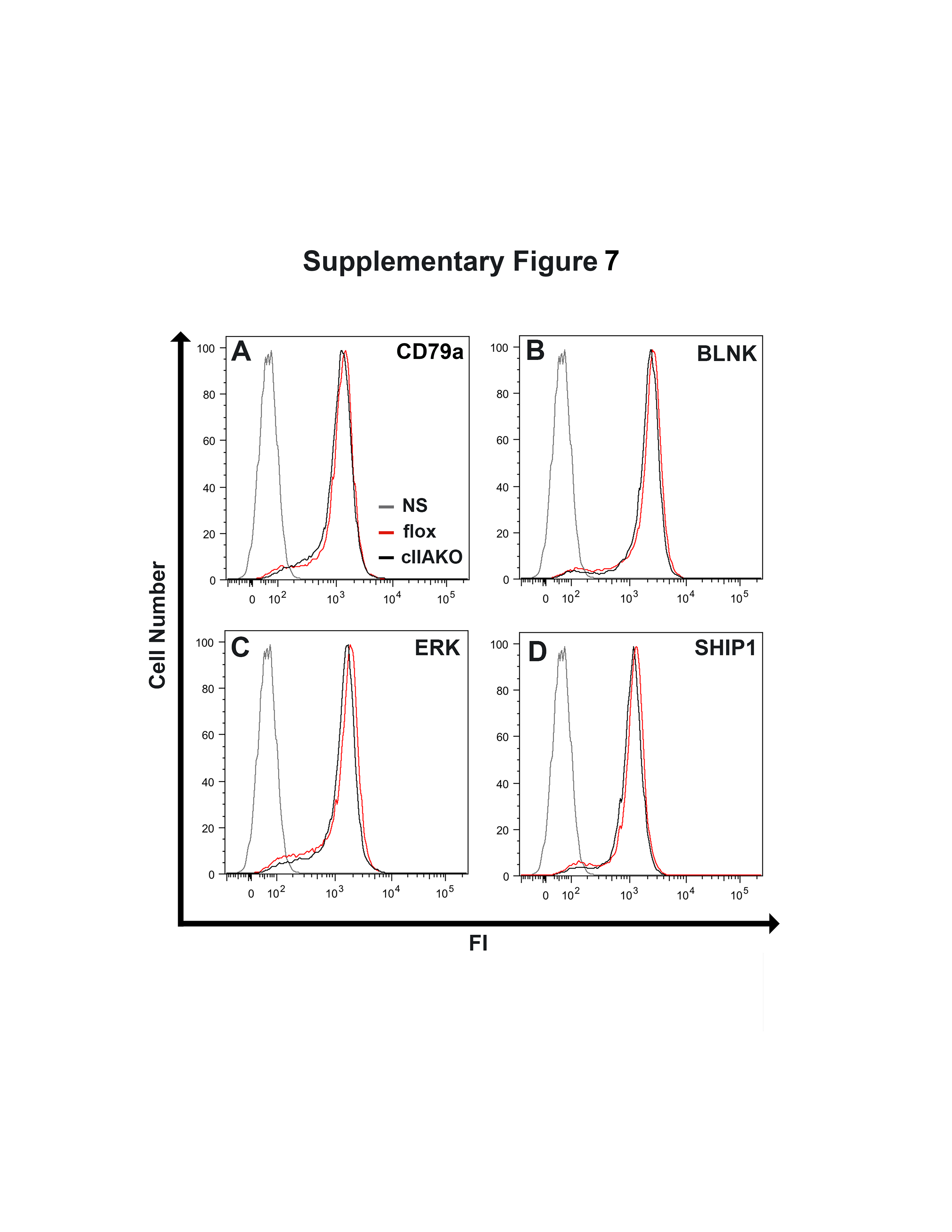

Supplement: Supplementary Figure 7 — Protein expression levels of signaling molecules in cIIAKO and floxed control B-cells. Floxed control and cIIAKO B-cells were fixed, permeabilized, labeled for CD79 (A), BLNK (B), Erk (C), and SHIP1 (D), and analyzed by flow cytometry. Shown are representative histograms from 3 independent experiments. ​ [file Image_7.tif]

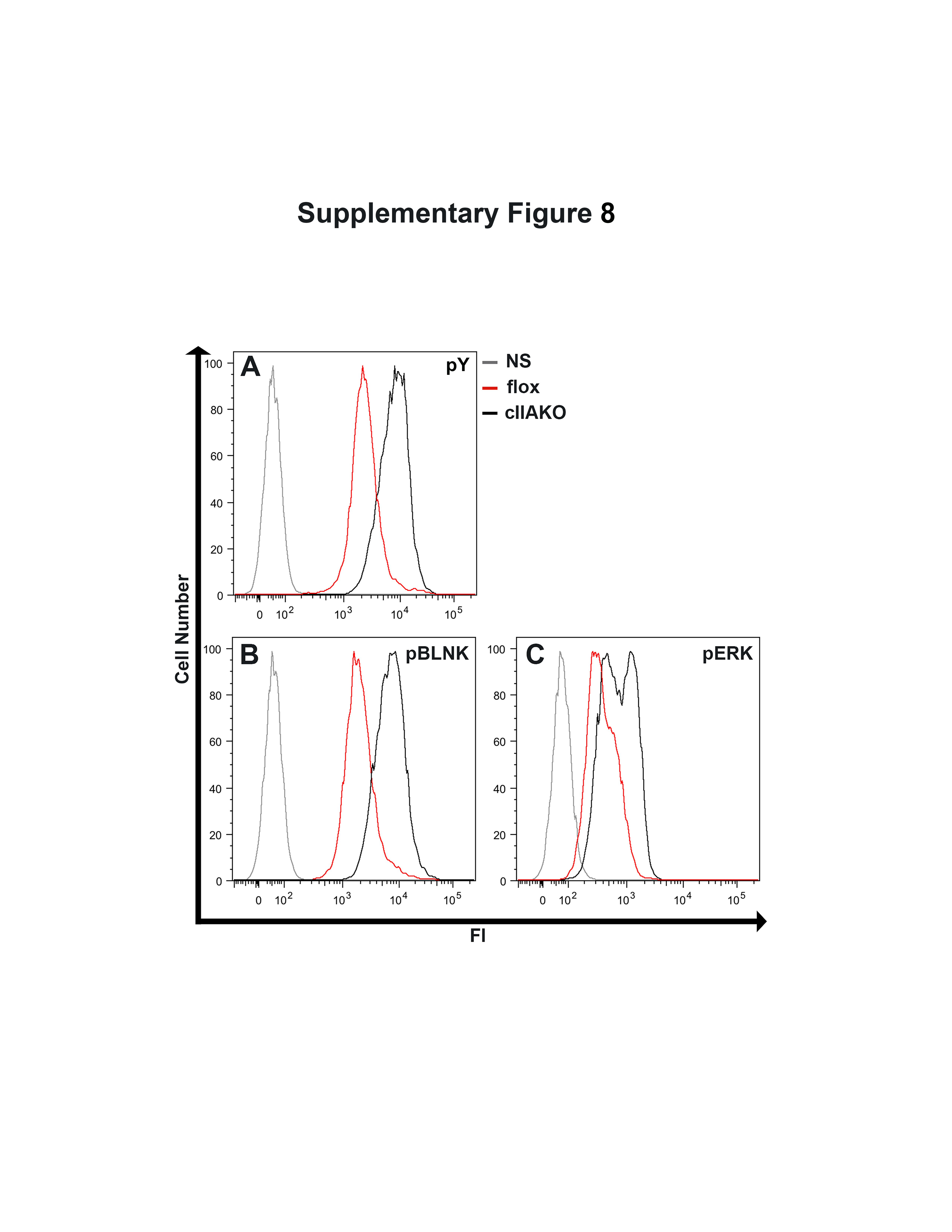

Supplement: Supplementary Figure 8 — BCR signaling in cIIAKO B-cells is enhanced in response to soluble stimulation. B-cells from floxed control and cIIAKO mice were activated with F(ab’)2 goat anti-mouse IgG+M, fixed, permeabilized, labeled for pY (A), pBLNK (B), and pErk (C), and analyzed by flow cytometry. Shown are representative histograms of pY (A) and pERK (C) at 5 min and pBLNK at 10 min (B) from three independent experiments. ​ [file Image_8.tif]
